# An ESIPT-Based Fluorescent Probe for Aqueous Cu^+^ Detection through Strip, Nanofiber and Living Cells

**DOI:** 10.3390/molecules28093725

**Published:** 2023-04-26

**Authors:** Zhao Cheng, Xilang Jin, Yinggang Liu, Lei Zheng, Hao He

**Affiliations:** 1 School of Pharmacy, Xi’an Medical University, Xi’an 710021, China; 2 School of Materials and Chemical Engineering, Xi’an Technological University, Xi’an 710032, China

**Keywords:** cuprous ion, dynamic imaging, strip, nanofiber, lysosome-targetable probe

## Abstract

Constructed on the benzothiazole-oxanthracene structure, a fluorescent probe RBg for Cu^+^ was designed under the ESIPT mechanism and synthesized by incorporating amide bonds as the connecting group and glyoxal as the identifying group. Optical properties revealed a good sensitivity and a good linear relationship of the probe RBg with Cu^+^ in the concentration range of [Cu^+^] = 0–5.0 μmol L^−1^. Ion competition and fluorescence-pH/time stability experiments offered further possibilities for dynamic Cu^+^ detection in an aqueous environment. HRMS analysis revealed a possible 1:1 combination of RBg and Cu^+^. In addition, colorimetric Cu^+^ detection and lysosome-targeted properties of the probe RBg were analyzed through RBg-doped PVDF nanofiber/test strips and RBg-Mito/Lyso trackers that were co-stained in living HeLa cells, enabling the probe’s future applications as real-time detection methods for dynamic Cu^+^ tracking in the lysosomes and Cu^+^ detection under diversified conditions.

## 1. Introduction

Copper [1,2], an abundantly existing element and active participant in the human body with the highest copper concentration occurring in the brain [3,4], has been proven to be connected with a number of neurodegenerative diseases [5,6]. On the one hand, commonly coexisting oxidation states of cuprous and copper ions in the biological systems are dynamic transforming and demonstrate an active oxidation–reduction reaction in participants. On the other hand, physiological copper signals are always accompanied by distribution, storage, transportation and other complex copper changes. Thus, if out of order [7], the dynamic changes of copper would induce abnormal accumulation or uncontrolled oxidation–reduction reactions [8], then trigger chain reactions that disrupt the natural balance, and would finally lead to neurodegenerative diseases such as Alzheimer’s and Parkinson’s Disease [9]. For a better understanding of the dynamic changes of Cu^+/2+^ in living systems, increasing copper-detecting techniques, especially those that could efficiently distinguish Cu^+^ from Cu^2+^ without sample damages, need to be developed.

From the pioneering studies of rhodamine Cu^2+^ probes by A. Czarnik [10], to P. Li [11] and C. Liu [12], to recent research by M. Tian [13], J. Qian [14] and L. Wang [15], numerous Cu^2+^-detecting methods have been developed. By contrast, probes for Cu^+^ detection are rare and still in high demand [16]. Besides the urgent need for the development of more Cu^+^ probes, increasing efforts should also be paid to solve the remained problems in practical Cu^+^ detections. In biological systems, a highly specific detection method should simultaneously demonstrate unique responses and good stabilities towards the target Cu^+^. Thus, the probe’s sensitivity and selectivity [17] towards Cu^+^ should be enhanced to overcome the coexistence of complex ions [18] and pH-changing environments [19,20]. Furthermore, optical Cu^+^ assessment would provide colorimetric information for practical probe-Cu^+^ detection processes.

Accordingly, the fluorescent probe RBg for Cu^+^ was constructed by incorporating amide bonds with the connecting group and the glyoxal identifying group, resulting in a benzothiazole-oxanthracene structure [21]. In aqueous solutions, the probe RBg demonstrated specific responses towards Cu^+^ over other metals (Cu^2+^ included), accompanied by a good quantitative relationship, good fluorescence–pH/time stabilities and remarkable changes in UV-Vis/fluorescent spectra of RBg-Cu^+^. Apart from aqueous Cu^+^ detection, optical Cu^+^ detection could be accomplished through RBg-doped PVDF nanofiber/test strips and in living cells, providing us with an instant and convenient Cu^+^ colorimetric method of great practical value.

## 2. Results

### 2.1. Structural Characterization

Constructed on the benzothiazole-oxanthracene structure, the Cu^+^ fluorescent probe RBg was synthesized by introducing a glyoxal fragment as the identifying section to rhodamine moiety. Structures of the probe RBg and related intermediates were characterized by ^1^H NMR and HRMS. The calculation and measurement results of MS were all matched, and characteristic ^1^H NMR chemical shifts were all located at the reasonable ppm range and in accordance with the target structures.

### 2.2. Spectroscopic Properties

#### 2.2.1. Selectivity and Competition

Considering the coexistence of complex ions for Cu^+^ in biological systems, accomplishing a specific detection of the target Cu^+^ by probe RBg is becoming more difficult. The selectivity of the probe RBg on Cu^+^ was evaluated in a PBS buffer solution (*V* (EtOH):*V* (H_2_O) = 1:99, pH = 7.4) with the concentration of RBg (10 μmol L^−1^) and metal ions (40 μmol L^−1^).

**Figure 1:**
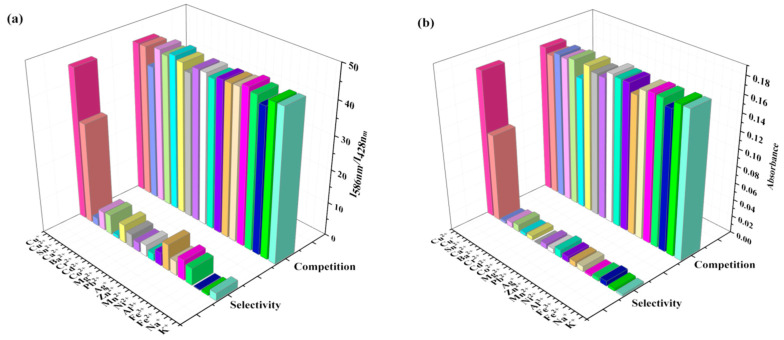
Selectivity and competition of probe RBg on Cu^+^ in Cu^+^ competing ion coexistence systems at the excitation wavelength of 380 nm: (**a**) fluorescence intensity ratio I_586_/I_428_ of the RBg-metal ion system; (**b**) absorbance at 563 nm of the RBg-metal ion system. The front row in (**a**,**b**): (**a**) fluorescence ratio I_586_/I_428;_ and (**b**) absorbance at 563 nm of probe RBg upon the addition of various metal ions (RBg: 10 μmol L^−1^, metal ions: 40 μmol L^−1^). The rear row in (**a**,**b**): a subsequent addition of 40 μmol L^−1^ Cu^+^ to the front-row represented solutions in (**a**,**b**).

At an excitation wavelength of 380 nm, the probe RBg displayed maximal fluorescent intensity at 428 nm, which could be attributed to the ESIPT process of benzothiazole moiety. Then, upon the addition of various metal ions, maximal fluorescence emission peaks of the RBg-Cu^+^ system declined at 428 nm, and a new emission at 586 nm significantly enhanced, which could be attributed to the ESIPT on-off and delocalized xanthene deformation–formation processes in the probe RBg before and after Cu^+^ combination. The obvious differences were illustrated by the fluorescence ratio I_586_/I_428_ in the front row of Figure 1a, and the absorbance at 563 nm of this RBg–metal ion system in Figure 1b. Only Cu^+^ could immediately trigger a significant optical enhancement at 586 nm for FS, and 563 nm for UV-Vis with a relatively low interference from Cu^2+^.

After a subsequent addition of 40 μmol L^−1^ Cu^+^ to the RBg-metal ion solutions (represented in the front row), the (a) fluorescence ratio I_586_/I_428_ and (b) absorbance at 563 nm of the resulting RBg–metal ion–Cu^+^ systems, are depicted in the rear row of Figure 1a,b. Compared with the front rows, in the complex coexistence system of Cu^+^ and other metal ions represented by the rear rows, the addition of Cu^+^ into the RBg–metal ion systems triggered significant increases of almost equal extent for every RBg–metal ion–Cu^+^ system, while the existence of other metal ions had nearly no competitions or influences on the specific optical responses of RBg-Cu^+^. The competition tests showed that the probe RBg was highly sensitive to Cu^+^, and predicted the future use of RBg for Cu^+^ detection in complex environments, which will not be affected by the tested background of other metal ions.

To evaluate the significant difference for the recognition properties of probe RBg in Figure 1, a standard *t*-test for the RBg–metal ion and RBg–metal ion–Cu^+^ systems was conducted, and the results with a confident level of 95% (alpha = 0.05) were shown in Table 1. In the case of fluorescent or UV-Vis analyses, significant differences were proven to exist between the RBg–metal ion and RBg–metal ion–Cu^+^ systems.

#### 2.2.2. Linear Relationship

**Figure 2:**
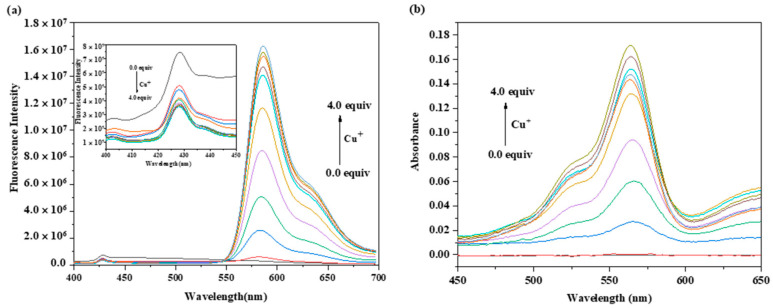
(**a**) Fluorescence and (**b**) UV-Vis spectra of probe RBg titrated with Cu^+^ at the excitation wavelength of 380 nm (RBg: 10 μmol L^−1^, Cu^+^: 0.0–4.0 equiv. to RBg).

Fluorescence and UV-Vis titration experiments were performed to analyze the quantitative relationship of probe RBg and Cu^+^. As a 10 μmol L^−1^ solution of RBg was gradually titrated with 0.0–4.0 equiv. Cu^+^, the enhancement for fluorescence (586 nm, Figure 2a) and UV-Vis absorbance (563 nm, Figure 2b) was resulted, accompanied by a contrary fluorescence decrease at 428 nm (Figure 2a, inset), which could be attributed to the ESIPT on–off and delocalized xanthene deformation–formation processes in probe RBg before and after Cu^+^ combination.

**Figure 3:**
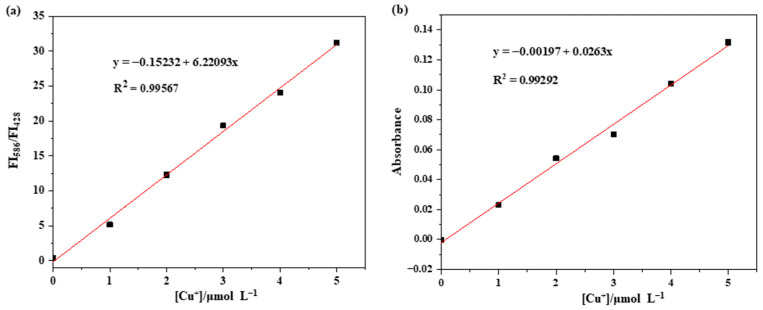
Linear relationships at the excitation wavelength of 380 nm: (**a**) fluorescence intensity ratio FI_586_/FI_428_ of the probe RBg versus [Cu^+^]; (**b**) absorbance at 563 nm of the probe RBg versus [Cu^+^].

Upon further analysis of the fluorescence of the probe and Cu^+^ system, a linear relationship was obtained in Figure 3a, indicating a highly quantitative detection of Cu^+^ by probe RBg in the concentration range of 0–5 μmol L^−1^ Cu^+^, as depicted. The linear response of RBg’s fluorescence intensity towards [Cu^+^] (0–5 μmol L^−1^) could be expressed by the following equation [22,23] through an analysis on the relationship of FI_586_/FI_428_ ratio (FI_586_, FI_428_: fluorescence intensity of the probe–Cu^+^ system at the wavelength of 586 and 428 nm) versus [Cu^+^]: y = −0.15232 + 6.22093x (*R*^2^ = 0.99567), and the corresponding detection limit of Cu^+^ by RBg was calculated to be 0.062 μmol L^−1^. A similar linear relationship of UV-Vis absorbance at 563 nm towards [Cu^+^] in the range of 0–5 μmol L^−1^ could be expressed as y = −0.00197 + 0.0263x (*R*^2^ = 0.99292), as depicted in Figure 3b.

For a better understanding on the accuracy of this Cu^+^ detection method, recovery tests for the RBg–Cu^+^ system was conducted, and the results are illustrated in Table 2. Whenever fluorescence or UV-Vis analyses were adopted, the average recoveries were maintained in the range of 100% ± 0.5%, with the relevant RSD values lower than 5%, which all combined to indicate the accuracy and reliability of the RBg–Cu^+^ quantitative method.

To ensure the robustness of this Cu^+^ analytical method by the probe RBg, interday measurements at 0.05 h, 0.1 h, 0.5 h, 1 h, 2 h and 4 h were conducted, giving the fluorescent intensity and absorbance values as x_1_–x_6_ in Table 3, resulting in RSD% lower than 5%. Furthermore, *t*-tests verified the statistically significant difference for the RBg–Cu^+^ system at varied Cu^+^ concentrations, as seen in Table 4.

#### 2.2.3. Influence Parameters

**Figure 4:**
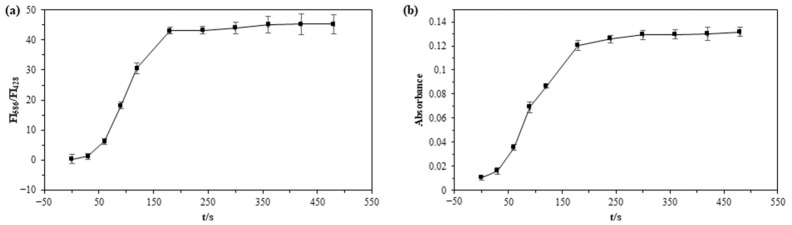
Time dependence of probe RBg and Cu^+^ at the excitation wavelength of 380 nm: (**a**) fluorescence intensity ratio FI_586_/FI_428_ versus time; (**b**) absorbance at 563 nm versus time (RBg: 10 μmol L^−1^, Cu^+^: 40 μmol L^−1^).

When applied to the detection of Cu^+^ in biological systems, fluorescence–time stabilities of the RBg–Cu^+^ system should be taken into consideration for possible time-lapse Cu^+^ detecting needs. At the time range of 0–500 s, the (a) fluorescence ratio FI_586_/FI_428_ and (b) absorbance at 563 nm of the RBg-Cu^+^ system showed an initial response time of about 170 s and remained steady for minutes (Figure 4), indicating a possible time-lapse fluorescence imaging application for dynamic Cu^+^ labelling and tracking in biological systems.

**Figure 5:**
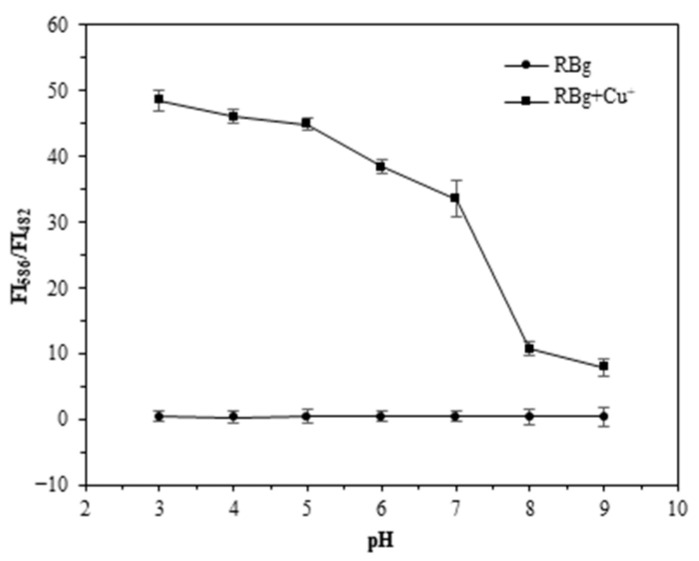
pH dependence of probe RBg in the absence and presence of Cu^+^.

As for the complex biological environment for applications of probe RBg, the fluorescence-pH stability was also examined. In comparison with the steady fluorescence intensity ratio FI_586_/FI_428_ of probe RBg at a varied pH region of 3.0 to 9.0, the fluorescence intensity ratio FI_586_/FI_428_ of RBg-Cu^+^ was initially kept relatively steady and gradually decreased (Figure 5), suggesting a pH range from 3.0 to 7.0, which is appropriate for the RBg-Cu^+^ combination system, and will satisfy most physiological pH environments for Cu^+^ detections [24].

### 2.3. Mechanism

**Figure 6:**
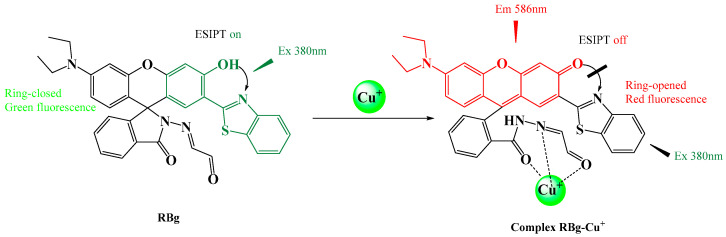
Proposed recognition mechanism of probe RBg to Cu^+^.

As illustrated in the fluorescence and UV-Vis titration spectra (Figure 2) before and after Cu^+^ combination, it can be inferred that an ESIPT on-off process in probe RBg was accompanied with the delocalized xanthene deformation–formation [25]. Specifically, an ESIPT on-off process was adopted by probe RBg to realize the recognition of Cu^+^ by relying on the intramolecular hydrogen bond between the phenolic hydroxyl group and the N atom of the thiazole ring in RBg (Figure 6). When the combination of RBg–Cu^+^ was accomplished through a spirocyclic-opening structural transformation, and accompanied with that were the delocalized xanthene formation and ESIPT off processes, resulting in a longer emission (red fluorescence, 586 nm) for the combined RBg–Cu^+^, due to the intramolecular hydrogen bond between the phenolic hydroxyl group and the N atom of the thiazole ring in RBg. In comparison with the 428 nm green fluorescence (in accordance with literatures) for the benzothiazole moiety at the state of ESIPT on, the ESIPT on-off processes, combined with the closing–opening spirocyclic ring, made the dynamic detection for Cu^+^ by RBg possible. Furthermore, release of the free and active proton in the phenolic hydroxyl group also contributed to the processes of ESIPT effect off and xanthene formation, leading to the recognition of RBg–Cu^+^.

Before the addition of Cu^+^ into RBg, the maximal fluorescence intensity in probe RBg emerged at 428 nm, which was consistent with the ESIPT-on and delocalized xanthene-deformation state, thus emitting the green fluorescence of the RBg ring-closed structure located at a short wavelength of 428 nm. When Cu^+^ was added into the RBg, the amide carbonyl group of the probe coordinated with Cu^+^, and thus RBg-Cu^+^ combined, the formation of the delocalized xanthene fragment not only inhibited the ESIPT process; meanwhile, a complex RBg–Cu^+^ with a better molecular rigid was also formed [25], indicating that the specific red fluorescence of the RBg ring-opened structure would be emitted, which was in accordance with the long-wavelength fluorescence emission enhancement at 586 nm in Figure 2a. As 0–40 μmol L^−1^ Cu^+^ was gradually added into RBg, the processes of ESIPT on-off and delocalized xanthene deformation–formation would be intensified, corresponding to the gradual fluorescence enhancements at 586 nm and the declines at 428 nm in Figure 2a.

**Figure 7:**
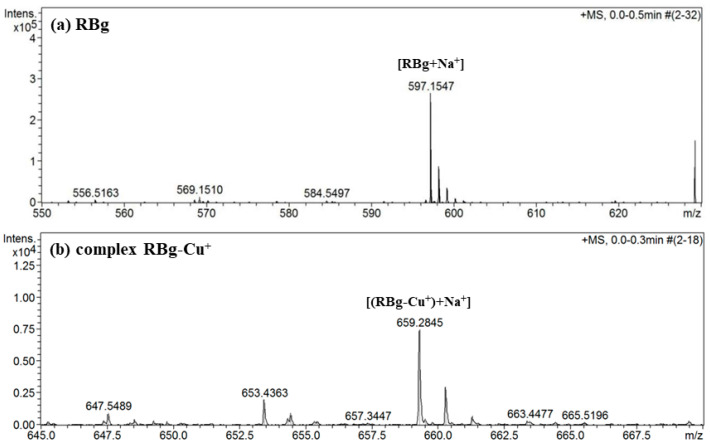
HRMS analysis of (**a**) RBg and (**b**) complex RBg–Cu^+^.

More importantly, HRMS analysis offered us a better understanding of the structural transformation from probe RBg to the coordination complex RBg-Cu^+^. After the addition of Cu^+^ into the probe RBg, a diagnostic peak at *m*/*z* = 659.2845 (probe RBg-Cu^+^) was obtained (Figure 7), which was in accordance with the calcd. *m*/*z* = 660.2857 for [M(RBg-Cu^+^)+Na^+^] and previously detected *m*/*z* = 597.1547 [M(RBg)+Na^+^], providing evidence for the 1:1 combination of RBg and Cu^+^ illustrated in the proposed mechanism (Figure 6).

### 2.4. Imaging Applications

#### 2.4.1. Detection and Imaging of Cu^+^ by RBg on Test Strips

**Figure 8:**
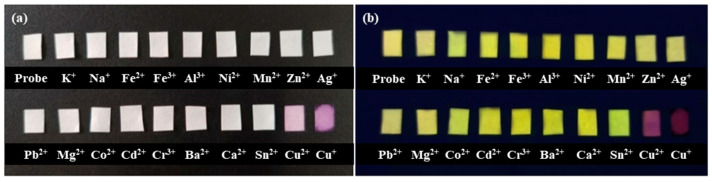
Photographs of the probe RBg-loaded test strips upon exposure to the aqueous solutions of various metal ions: (**a**) under ambient light; (**b**) under a hand-held UV lamp at 365 nm.

To explore the sensing and imaging behavior of the synthesized probe, RBg-loaded test strips were immersed into a series of one metal ion-containing aqueous solutions (containing Cu^+^ and other 18 metal ions, 5 μmol L^−1^ for Cu^+^ and 100 μmol L^−1^ for others) for 10 min and then imaged, respectively. As seen in Figure 8, whether the resulted test strips were located (a) under ambient light or (b) under the 365 nm ultraviolet lamp, only the Cu^+^ solution demonstrated obvious color changes and could be simultaneously distinguished by naked eyes and ultraviolet [26,27], presenting a potentially quick and compatible Cu^+^ colorimetric method sensitively happening on the probe-loaded test strips.

#### 2.4.2. Detection and Imaging of Cu^+^ by RBg on Solid-State Nanofibers

**Figure 9:**
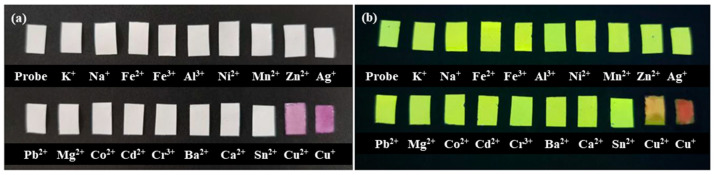
Photographs of probe RBg–PVDF composite nanofibers upon exposure to the aqueous solutions of various metal ions: (**a**) under ambient light; (**b**) under a hand-held UV lamp at 365 nm.

For the detection and imaging of Cu^+^ by RBg on solid-state, RBg-PVDF composite nanofibers were prepared and immersed into Cu^+^ (5.0 μmol L^−1^)/other cation solutions (100 μmol L^−1^), respectively, and then imaged under ambient light or under the 365 nm ultraviolet lamp (Figure 9). Not surprisingly, only Cu^+^ induced a significant colorless-to-red colorimetric and green-to-red fluorescence change, accomplishing a simultaneous naked-eye Cu^+^ detection under ambient light and spectroscopic Cu^+^ detection under a UV lamp.

#### 2.4.3. Detection and Imaging of Cu^+^ by RBg in Living HeLa Cells

**Figure 10:**
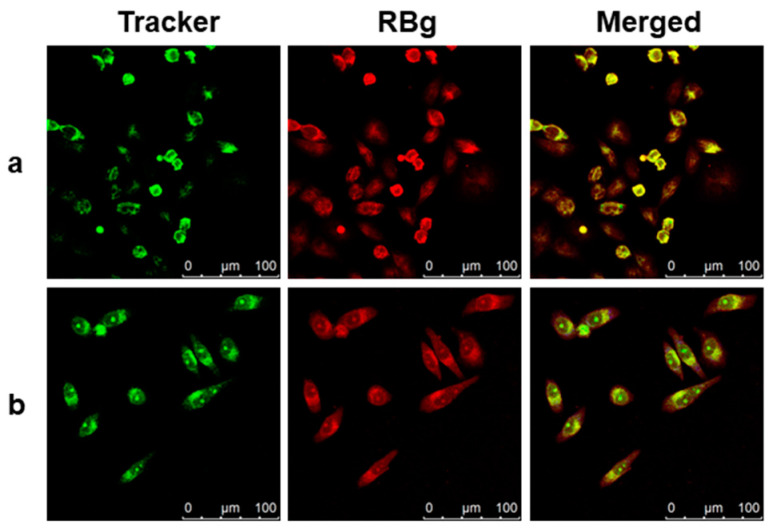
Co-staining fluorescence imaging of RBg + Cu^+^ and organelle-targeting trackers in HeLa: (**a**) the fluorescent image of HeLa costained with 1 μmol L^−1^ RBg, 1 μmol L^−1^ Cu^+^ and 100 nmol L^−1^ LysoTracker Green; (**b**) the fluorescent image of HeLa costained with 1 μmol L^−1^ RBg, 1 μmol L^−1^ Cu^+^ and 100 nmol L^−1^ MitoTracker Green. Red channel: λ_ex_ = 559 nm, λ_em_ = 688–788 nm. Green channel: λ_ex_ = 488 nm, λ_em_ = 511–539 nm.

Through the bioimaging experiment for dynamic Cu^+^ tracking in HeLa cells, the accumulated areas of RBg in the cytoplasm were analyzed. By incorporating commercially available localization reagents, such as Mito-Tracker Green (Mito) and Lyso-Tracker Green (Lyso), the co-staining experiments of RBg–Mito/Lyso were performed, and lysosome targeting properties of the synthesized RBg were revealed. As displayed in Figure 10a, RBg + Cu^+^ and the lysosome-targeting reagent Lyso-Tracker Green, demonstrated a good overlap that presented large yellow patches. In contrast with Figure 10a, RBg + Cu^+^ and the mitochondria-targeting reagent MitoTracker Green, provided a poor overlap which is shown as the red/green patches in Figure 10b. Consequently, the co-staining bioimaging indicated that RBg could be used as an efficient lysosome-targetable probe for dynamic Cu^+^ imaging in living cells, providing potential tools for monitoring the function of lysosomes in the autophagy processes.

## 3. Discussion

With the structure characterized by HRMS/^1^H NMR and optical properties analyzed by UV-Vis/fluorescence/imaging, a fluorescent probe RBg was proven quite useful for the colorimetric and dynamic detection of aqueous Cu^+^. On the one hand, highly specific responses to Cu^+^ over other metal ions were accomplished by probe RBg with 1:1 association stoichiometry of RBg-Cu^+^ and a low detection limit of [Cu^+^] = 0.062 μmol L^−1^. On the other, HRMS analysis offered us sufficient information on the combination mechanism. The structural transformation of the probe in the recognition process of Cu^+^ was also studied and possible recognition sites in RBg were labelled. In addition, the combination of RBg and Cu^+^ emitted significant optical signals in RBg-doped PVDF nanofibers and test strips, which targeted the instant labelling and detection of dynamic Cu^+^, unique to the aqueous environment. Apart from the good fluorescence stability at a relatively wide physiological pH range and in a considerably great time span, probe RBg also demonstrated lysosome-targeted properties in the co-staining experiment in HeLa cells, which combined the predicted probe RBg appropriate for the biological applications under complex environments.

## 4. Materials and Methods

### 4.1. Reagents and Equipments

^1^H NMR spectra were recorded on INOVA-400 spectrometer (Varian, Palo Alto, CA, USA) at 400 MHz and chemical shifts were reported relative to internal standard tetramethylsilane (TMS). Fluorescent spectra were measured with F-4500 fluorescence spectrophotometer (HITACHI, Tokyo, Japan). UV-Vis spectra were measured on UV-2550 spectrometer (SHIMADZU, Kyoto, Japan). Cell-imaging experiments were performed using FV1000 TY1318 laser scanning confocal microscope (OLYMPUS, Tokyo, Japan). MS analyses were performed on micro TOF-Q II mass spectrophotometer (Bruker, Billerica, MA, USA) at the positive mode and in a wide range of 500–10,000 *m*/*z* with the following acquisition parameters: Source Type (ESI); Ion Polarity (Positive); Set Nebulizer (0.3 Bar); Focus (Active); Set Capillary (4500 V); Set Dry Heater (180 °C); Scan Range (500–10,000 *m*/*z*); Set End Plate Offset (−500 V); Set Dry Gas (4.0 L/min); Set Collision Cell RF (1000.0 Vpp); and Set Divert Valve (Source).

All the reagents and solvents used for synthesis were commercially available and used without further purification, unless otherwise noted. The reaction process was monitored by thin-layer chromatography (TLC) on silica gel GF254. The products were purified by column chromatography on Merck silica gel (250–400 mesh ASTM). Phosphate buffered saline (PBS, pH = 7.4) was purchased from Sinopharm Chemical Reagent Company, Shanghai, China. Double distilled water was used throughout the process of solution preparing and spectroscopic testing. Solutions of metal ions were prepared from their nitrate and chloride salts, CuCl, CuCl_2_, SnCl_2_, CaCl_2_, BaCl_2_, Cr(NO_3_)_3_, CdCl_2_, CoCl_2_, MgCl_2_, PbCl_2_, AgNO_3_, ZnCl_2_, MnCl_2_, NiCl_2_, AlCl_3_, FeCl_3_, FeCl_2_, NaCl and KCl.

### 4.2. Synthesis of Probe RBg

**Scheme 1:**
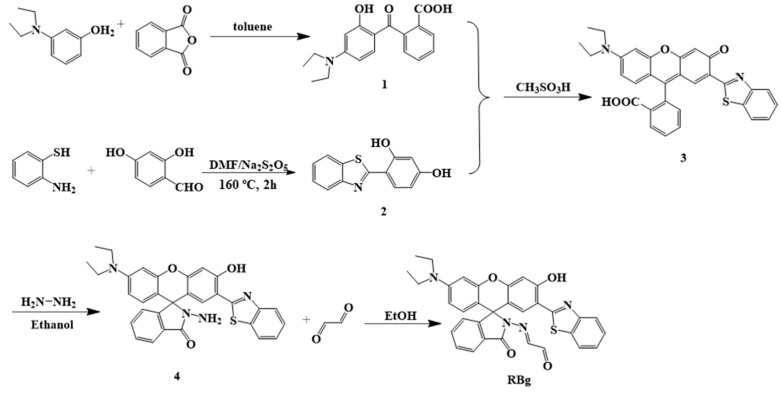
Synthetic route of probe RBg.

The fluorescent probe RBg was synthesized by incorporating amide bonds as the connecting group and glyoxal identifying group into a benzothiazole-oxanthracene structure, as shown in Scheme 1. The hydrogen information of RBg was characterized by ^1^H NMR, and molecular weights were analyzed by HRMS. Additionally, structures and properties were further confirmed by UV-Vis and fluorescent analyses.

Compounds **1**–**4** in Scheme 1 were synthesized according to the reported methods and the structural characterizations of compounds **1**–**2**, which were in accordance with the literature reports [28]. The obtained compd. **4** (0.534 g, 1.00 mmol) and glyoxal (0.058 g, 1.10 mmol) were then dissolved in ethanol (25 mL), and the mixture reacted in an N_2_ atmosphere at 80 °C for 24 h. After the reaction, the mixture was concentrated in vacuo and subsequently purified by column chromatography (silica gel, *V* (dichloromethane):*V* (hexane) = 2:1 as eluent) to afford the probe RBg.

Compounds **3**: 0.5075 g, red solid; yield 45.6%. ^1^H NMR (400 MHz, Chloroform-d) δ: 1.22 (t, *J* 7.0 Hz, 6H), 3.40 (q, *J* 7.1 Hz, 4H), 5.32 (s, 1H), 6.45 (m, 3H), 6.95 (s, 1H), 7.07 (s, 1H), 7.29 (d, *J* 3.0 Hz, 1H), 7.37 (t, *J* 7.6 Hz, 1H), 7.47 (m, 1H), 7.69 (m, 2H), 7.80 (d, *J* 7.9 Hz, 1H), 7.96 (d, *J* 8.1 Hz, 1H), 8.10 (m, 1H). MS: calcd. for *m*/*z* = 521.1465 [M+H^+^], found 521.1605.

Compounds **4**: 1.5801 g, red solid; yield 43.7%. ^1^H NMR (400 MHz, Chloroform-d) δ: 1.22 (t, *J* 7.0 Hz, 6H), 3.38 (q, *J* 7.1 Hz, 4H), 5.32 (s, 1H), 6.36 (dd, *J* 8.9, 2.6 Hz, 1H), 6.49 (m, 2H), 6.94 (s, 1H), 6.98 (s, 1H), 7.15 (s, 1H), 7.18 (dd, *J* 6.1, 2.5 Hz, 1H), 7.38 (t, *J* 7.6 Hz, 1H), 7.48 (t, *J* 7.7 Hz, 1H), 7.55 (m, 2H), 7.75 (s, 1H), 7.81 (d, *J* 8.0 Hz, 1H), 7.93 (d, *J* 8.2 Hz, 1H), 8.03 (dd, *J* 6.3, 2.6 Hz, 1H). MS: calcd. for *m*/*z* = 535.1726 [M + H^+^], found 535.1840.

Probe RBg: 0.3590 g, yellow solid; yield 60.6%. ^1^H NMR (400 MHz, DMSO-d_6_) δ: 1.11 (m, 6H), 3.46 (t, *J* 7.0 Hz, 5H), 6.51 (m, 3H), 7.02 (s, 1H), 7.25 (d, *J* 7.5 Hz, 1H), 7.40 (d, *J* 8.1 Hz, 1H), 7.47 (d, *J* 1.3 Hz, 1H), 7.63 (s, 1H), 7.73 (ddd, *J* 14.6, 7.4, 1.3 Hz, 2H), 7.82 (d, *J* 7.4 Hz, 1H), 7.96 (d, *J* 8.1 Hz, 1H), 8.07 (d, *J* 8.0 Hz, 1H), 8.12 (d, *J* 7.5 Hz, 1H), 9.27 (d, *J* 7.4 Hz, 1H). MS: calcd. for *m*/*z* = 597.1675 [M + Na^+^], found 597.1547.

### 4.3. Spectroscopic Analysis

Stock solutions (100 μmol L^−1^) of probe RBg, Cu^+^, Cu^2+^, Sn^2+^, Ca^2+^, Ba^2+^, Cr^3+^, Cd^2+^, Co^2+^, Mg^2+^, Pb^2+^, Ag^+^, Zn^2+^, Mn^2+^, Ni^2+^, Al^3+^, Fe^3+^, Fe^2+^, Na^+^ and K^+^ were prepared in EtOH-H_2_O (*V* (EtOH):*V* (H_2_O) = 1:99, PBS, pH = 7.4). When used for spectroscopic tests, the stock solutions were usually diluted with EtOH-H_2_O (*V* (EtOH):*V* (H_2_O) = 1:99, PBS, pH = 7.4) to 10 μmol L^−1^, unless noted. All the spectroscopic measurements were performed at least in triplicate and averaged.

### 4.4. Colorimetric Imaging

For the detection and imaging of Cu^+^ by RBg on test strips and solid-state nanofibers, test strips for Cu^+^ were prepared by dipping filter paper into the PBS buffer solution of probe RBg for 2 h (*V* (EtOH):*V* (H_2_O) = 1:99, pH = 7.4), and composite nanofibers that contained probe RBg and polyvinylidene fluoride (PVDF M_w_ = 30,000, *m* (RBg):*m* (PVDF) = 1:1000, DMF solution) as the matrix were prepared through the electrospinning technique. When used for test strip imaging and nanofiber imaging, the RBg-loaded test strips and RBg-PVDF composite nanofibers were then immersed into a targeted ion-containing aqueous solution and imaged under ambient light or under the 365 nm ultraviolet lamp.

### 4.5. Bioimaging

HeLa cells were cultured in 10% FBS-containing DMEM at 37 °C in the humidified atmosphere with 5% CO_2_. After 2 h, the growth medium was removed, and the cells were first washed with DMEM and incubated with 10 μmol L^−1^ RBg and 100 nmol L^−1^ Mito/LysoTracker for 30 min at 37 °C, then washed three times with PBS, costained with 10 μmol L^−1^ Cu^+^ for 30 min and imaged [26,27].

## Figures and Tables

**Table 1:** *t*-tests for RBg–metal ion and RBg–metal ion–Cu^+^ systems.

***t*** **-Test in Fluorescent Analyses (conc.: 10 μmol L^−1^ RBg, 40 μmol L^−1^ Metal Ions/Cu^+^, x: FI_586_/FI_428_)**							
$\bar{x}$ **of RBg-Metal Ion**	$\bar{x}$ **of RBg–Metal Ion–Cu^+^**	***p*** **Value**	***t***	**d*f***	***R*** **^2^**	**95% Confidence Interval**	**Significantly Different (*p* < 0.05)**
0.004976	0.1681	<0.0001	122.5	32	0.9979	0.1604–0.1658	yes
***t*** **-Test in UV-Vis Analyses (conc.: 10 μmol L^−1^ RBg, 40 μmol L^−1^ Metal Ions/Cu^+^, x: Absorbance)**							
$\bar{x}$ **of RBg–Metal Ion**	$\bar{x}$ **of RBg–Metal Ion–Cu^+^**	***p*** **Value**	***t***	**d*f***	***R*** **^2^**	**95% Confidence Interval**	**Significantly Different (*p* < 0.05)**
3.761	44.68	<0.0001	64.29	32	0.9923	39.62–42.21	yes

**Table 2:** Recovery tests for the RBg–Cu^+^ system.

**FI_586_/FI_428_**					
**Content** **(μmol L** **^−1^** **)**	**Added** **(μmol L** **^−1^** **)**	**Measured** **(μmol L** **^−1^** **)**	**Recovery** **(%)**	**Average Recovery** **(%)**	**RSD** **(%)**
1	1	2.0332	101.66%	99.31%	2.192%
1	1	2.0604	103.02%		
1	1	1.9470	97.35%		
1	2	2.9187	97.29%		
1	2	2.9496	98.32%		
1	2	3.0051	100.17%		
1	4	4.9279	98.56%		
1	4	5.0325	100.65%		
1	4	4.8370	96.74%		
**Absorbance**					
**Content** **(μmol L** **^−1^** **)**	**Added** **(μmol L** **^−1^** **)**	**Measured** **(μmol L** **^−1^** **)**	**Recovery** **(%)**	**Average Recovery** **(%)**	**RSD** **(%)**
1	1	1.9577	97.88%	100.25%	2.094%
1	1	200.14	100.07%		
1	1	208.56	104.28%		
1	2	3.0328	101.09%		
1	2	2.9613	98.71%		
1	2	2.9415	98.05%		
1	4	4.9723	99.45%		
1	4	5.0160	100.32%		
1	4	5.1220	102.44%		

**Table 3:** The interday measurements for the RBg–Cu^+^ system.

**RSD in RBg–Cu^+^ Fluorescent Analyses (conc.: 10 μmol L^−1^ RBg, 40 μmol L^−1^ Cu^+^, x: FI_586_/FI_428_)**							
**x_1_**	**x_2_**	**x_3_**	**x_4_**	**x_5_**	**x_6_**	$\bar{x}$ **± *s***	**RSD%**
43.0265	43.2000	44.0230	45.1236	45.2350	45.2057	44.3023 ± 1.0278	2.3198%
**RSD in RBg–Cu^+^ UV-Vis Analyses (conc.: 10 μmol L^−1^ RBg, 40 μmol L^−1^ Cu^+^, x: Absorbance)**							
**x_1_**	**x_2_**	**x_3_**	**x_4_**	**x_5_**	**x_6_**	$\bar{x}$ **± *s***	**RSD%**
0.1204	0.1256	0.1293	0.1295	0.1300	0.1317	0.1278 ± 0.004118	3.2232%

**Table 4:** *t*-tests for the RBg-Cu^+^ system with varied Cu^+^ concentrations.

***t*** **-Tests in RBg–Cu^+^ Fluorescent Analyses (conc.: 10 μmol L^−1^ RBg, Varied Cu^+^, x: FI_586_/FI_428_)**						
**1 μmol L^−1^ Cu^+^**	**3 μmol L^−1^ Cu^+^**	**5 μmol L^−1^ Cu^+^**	***F***	***p*** **Value**	**Statistically Significant (*p* < 0.05)**	***R*** **^2^**
x_1_ 5.0413	x_1_ 19.5498	x_1_ 30.9045	4810	0.0002	yes	0.9996
x_2_ 5.3454	x_2_ 18.9978	x_2_ 31.5612				
x_3_ 5.0779	x_3_ 19.4712	x_3_ 31.1623				
***t*** **-Tests in RBg–Cu^+^ UV-Vis Analyses (conc.: 10 μmol L^−1^ RBg, Varied Cu^+^, x: Absorbance)**						
**1 μmol L^−1^ Cu^+^**	**3 μmol L^−1^ Cu^+^**	**5 μmol L^−1^ Cu^+^**	***F***	***p*** **Value**	**Significant Diff. among Means (*p* < 0.05)**	***R*** **^2^**
x_1_ 0.0222	x_1_ 0.0724	x_1_ 0.1305	1032	<0.0001	yes	0.9971
x_2_ 0.0235	x_2_ 0.0698	x_2_ 0.1369				
x_3_ 0.0234	x_3_ 0.0685	x_3_ 0.1279				

## Data Availability

The data presented in this study are available in the article.
